# The association between subclinical hypothyroidism and erectile dysfunction

**DOI:** 10.12669/pjms.343.14330

**Published:** 2018

**Authors:** Dawei Chen, Yuerong Yan, Hui Huang, Qiang Dong, Haoming Tian

**Affiliations:** 1 Dawei Chen, MD. West China Hospital, Sichuan University, 610041 Chengdu, Sichuan, China; 2 Yuerong Yan, MD. West China Hospital, Sichuan University, 610041 Chengdu, Sichuan, China; 3 Prof. Hui Huang. West China Hospital, Sichuan University, 610041 Chengdu, Sichuan, China; 4 Prof. Qiang Dong. Department of Urology, West China Hospital, Sichuan University, 610041 Chengdu, Sichuan, China; 5 Prof. Haoming Tian. West China Hospital, Sichuan University, 610041 Chengdu, Sichuan, China

**Keywords:** Erectile dysfunction, Subclinical hypothyroidism, Euthyroidism

## Abstract

**Objectives:**

Erectile dysfunction (ED) is highly prevalent among males, and hypothyroidism is previously reported to be related with ED. However there have been rare studies to investigate the association between subclinical hypothyroidism (SCH) and ED, hence our objective was to fill this gap.

**Methods:**

ED patients who visited the Urology Outpatients Clinic owere recruited consecutively, and males from the Health Manage Center were included as the controls. Serum thyroid and sexual hormones were estimated, and the International Index of Erectile Function (IIEF-5) questionnaires were evaluated as well. Subjects with normal sexual hormones were included for statistical analysis.

**Results:**

One hundred nine ED patients and 32 healthy controls were included in this study. The ratio of SCH and euthyroidism in ED males was 29.36% and 66.06% respectively. The IIEF-5 scores in ED patients with SCH were significantly lower than the controls with euthyroidism (P<0.05). The serum concentrations of TSH and prolactin were significantly higher and free thyroxine lower in ED patients with SCH when compared with the controls with euthyroidism (all p<0.05), and no significant differences of estradiol and total testosterone were found between those two groups. However the IIEF-5 scores were not significantly different between males with SCH and euthyroidism among ED patients (P>0.05).

**Conclusions:**

SCH is common in ED patients and may be associated with ED, whereas the severity of ED is not related to SCH. Screening for thyroid dysfunction in men presenting with ED is recommended.

## INTRODUCTION

Erectile dysfunction (ED) is defined by the consistent or recurrent inability to attain or maintain penile erection adequate for sexual intercourse.^1^ Epidemiological studies have suggested that more than 30 million males in United States are affected by ED.^2^ QJ Zhang and his colleagues reported that the prevalence of ED is 26.1% in the three major cities of Beijing, Chongqing and Guangzhou in China.^3^ Penile erection is a complex process involving psychogenic and hormonal input.^4^ Endocrine disorders, hypogonadism, hyperprolactinemia and thyroid diseases, are common risk factors of ED.^5^,^6^ Previous studies have reported that in overt hypothyroidism, the ratio of ED reach up to 52.1% and moreover overt thyroid function failure adversely affects erectile function among males.^7^,^8^

Subclinical hypothyroidism (SCH) is defined as mild thyroid function failure and diagnosed by elevated thyroid stimulating hormone (TSH) with normal free thyroxine (FT4) concentration.^9^ SCH has a possibility to progress to overt hypothyroidism. Epidemiological studies showed that the prevalence of SCH was much higher than overt hypothyroidism (3.4%~5.8% vs. 0.3%~0.7%).^10^,^11^ However there have been rare studies reported the association between mild thyroid failure and ED. Hence the objective of this study was to investigate the relationship between SCH and ED in males with normal sexual hormones levels and elucidate the effect of SCH on ED in further.

## METHODS

This cross-sectional study was conducted in West China Hospital of Sichuan University located in Chengdu, China. Consecutive patients with ED who visited the Outpatients of Urology Clinic between November 2012 and December 2013 were included. The inclusion criteria were ED patients aged 18 to 65 years with normal sexual hormones. The exclusion criteria were individuals with previous or current overt hypothyroidism or hyperthyroidism; individuals with a history of neck surgery; individuals with a history of ^131^I treatment or radiotherapy; individuals suffering from diabetes mellitus, renal insufficiency or any other serious systemic disease or chronic wasting disease; individuals treated with sexual hormones or PDE5-inhibitors within three months.

In addition, males aged 18 to 65 years and with normal serum thyroid hormones from the Health Manage Center were included as controls and were screened with the same exclusion criteria mentioned above. All participants provided written informed consent, and the study was approved by the Biomedical Ethics Committee of West China Hospital, Sichuan University, China.

### Measurements

Serum levels of thyroid stimulating hormone (TSH), free triiodothyronine (FT3), free thyroxine (FT4), estradiol (E2), prolactin (PRL) and total testostrone (TT) were measured by electrochemiluminescence immunoassay (Roche Kit, Cobas-e601 analysertotal). The normal reference ranges were as follows: TSH 0.27-4.2 mU/L, FT3 3.60-7.50 pmol/L, FT4 12.0-22.0 pmol/L, TT 2.49-8.36 ng/mL, E2 7.63-42.59 pg/mL, and PRL 4.6-21.4 ng/mL. Subclinical hypothyroidism (SCH) was defined by TSH >4.2mU/L and FT3 and FT4 within the reference range. Normal thyroid function (euthyroidism) was based on the levels of TSH, FT3 and FT4 within the reference range.

International Index of Erectile Function (IIEF-5) questionnaire was used to diagnose ED if IIEF-5 score ≤ 21 and the Chinese version of IIEF-5 have been extensively used in patients and general populations with good reliability and validity.^12^

### Statistical Analysis

Data processing was performed with SPSS version 17.0. The independent sample t-test and Chi-square test (fisher exact analysis) was used for comparisons of quantitative and qualitative variables between groups, respectively. All quantitative data were shown as mean ± SD. A *p*-value of < 0.05 was considered as statistically significant.

## RESULTS

### The baseline characteristics of ED subjects

A total of 109 ED patients with normal sexual hormones were included in this study. The ratio of SCH in which was 29.36% (32/109, three with TSH >10.0 mU/L), and the ratio of euthyroidism was 66.06% (72/109) respectively. The mean age and the ratio of smoking/drinking status showed no significant differences between ED with SCH and ED with euthyroidism (all p>0.05, Table-I).

**Table-I:** The baseline characteristics between males with SCH and euthyroidism among ED patients.

	ED with SCH (n=32)	ED with Euthyroidism (n=72)	
Age (y)	32.44 ± 5.84	34.76 ± 7.69	P>0.05
Smoking			P>0.05
Current smoking	12	28	
Never/previous smoking	20	44	
Drinking			P>0.05
Constantly	4	5	
Occasionally	28	67	

ED: Erectile Dysfunction, SCH: Subclinical hypothyroidism.

### The IIEF-5 scores, thyroid and sexual hormone levels of males with SCH and euthyroidism among ED patients

As shown in Table-II, the IIEF-5 scores were not significantly different between those two groups (P>0.05). The serum concentrations of TSH, and PRL were significantly increased in ED subjects with SCH than ED patients with euthyroidism, and FT4 was significantly decreased in ED with SCH when compared with those with euthyroidism (all P<0.05, Table-II). However there were no significant differences between serum levels of FT3,E2 and TT between the two groups (all P>0.05, Table-II).

**Table-II:** The IIEF-5 scores and concentrations of thyroid and sexual hormones between males with SCH and euthyroidism among ED patients.

	ED with SCH (n=32)	ED with Euthyroidism (n=72)	
IIEF-5 scores	11.31 ± 4.47	11.63±5.07	P>0.05
TSH (mU/l)	6.05 ± 2.17	2.12±0.89	P<0.05
FT3 (pmol/l)	5.49 ± 0.50	5.55 ± 0.58	P>0.05
FT4 (pmol/l)	15.73 ± 1.83	16.72 ± 1.88	P<0.05
TT (ng/ml)	5.00 ± 1.64	4.72 ± 1.12	P>0.05
E2 (pg/ml)	32.88 ± 9.59	31.36 ± 6.70	P>0.05
PRL (ng/ml)	11.48 ± 3.74	9.20 ± 3.12	P<0.05

IIEF-5: International Index of Erectile Function questionnaire, ED: Erectile dysfunction,

SCH: Subclinical hypothyroidism, TSH: Thyroid stimulating hormone, FT3: Free triiodothyronine,

FT4: Free thyroxine, TT: Total testostrone, E2: Estradiol, PRL: prolactin

### The baseline characteristics of ED with SCH and the controls with euthyroidism

Further 32 euthyroidism were included as the controls to compare with ED males with SCH. As shown in table-III, the mean age, BMI and the ratio of smoking/drinking status showed no significant differences between ED males with SCH and the controls with euthyroidism (all p>0.05).

**Table-III:** The baseline characteristics between ED with SCH and the controls with euthyroidism.

	ED with SCH (n=32)	Controls with Euthyroidism (n=32)	P
Age (y)	32.44 ± 5.84	32.44 ± 5.74	>0.05
BMI (kg/m^2^)	23.97 ± 2.90	24.22 ± 2.86	>0.05
Smoking			>0.05
Current smoking	12	11	
Never/previous smoking	20	21	
Drinking			>0.05
Constantly	4	5	
Occasionally	28	27	

ED: Erectile dysfunction, SCH: Subclinical hypothyroidism, BMI: Body Mass Index.

### The IIEF-5 scores and thyroid hormone levels of ED with SCH and the controls with euthyroidism

The IIEF-5 scores in ED patients with SCH were significantly lower than the controls with euthyroidism (P<0.05) as shown in Table-IV. There were no significant differences in serum levels of FT3, E2 and TT between ED patients with SCH and the controls with euthyroidism (all P>0.05, Table-IV), while the serum concentrations of TSH and PRL were significantly higher, and FT4 was significantly decreased in ED males with SCH than euthyroidism controls (all P<0.05, Table-IV).

**Table-IV:** The IIEF-5 scores and concentrations of thyroid and sexual hormones between ED with SCH and the controls with euthyroidism.

	ED with SCH (n=32)	Controls with Euthyroidism (n=32)	
IIEF-5 scores	11.31 ± 4.47	23.38 ± 0.94	P<0.05
TSH (mU/l)	6.05 ± 2.17	2.40±1.17	P<0.05
FT3 (pmol/l)	5.49 ± 0.50	5.55±0.47	P>0.05
FT4 (pmol/l)	15.73 ± 1.83	17.90±1.58	P<0.05
TT (ng/ml)	5.00 ± 1.64	4.80±1.26	P>0.05
E2 (pg/ml)	32.88 ± 9.59	33.03±6.44	P>0.05
PRL (ng/ml)	11.48 ± 3.74	8.96±2.45	P<0.05

IIEF-5: International Index of Erectile Function questionnaire, ED: Erectile dysfunction,

SCH: Subclinical hypothyroidism, TSH: Thyroid stimulating hormone, FT3: Free triiodothyronine,

FT4: Free thyroxine, TT: Total testostrone, E2: estradiol, PRL: Prolactin.

## DISCUSSION

After excluding males with dis-normal sexual hormones, this study found that the ratio of SCH was 29.36% in males with erectile dysfunction, and that was much higher than the 5.43% prevalence of SCH in males in the same area as the previous epidemiological study.^13^ ED is extremely common in males with dysthyroidism.^7^ Krysiak R and his colleagues reported that men with subclinical hypothyroidism are characterized with erectile dysfunction when compared with healthy euthyroid males.^14^ We found similar result that ED patients with SCH had significant lower IIEF-5 scores when compared with healthy euthyroid males. Those results indicated that mild thyroid failure may be related to erectile dysfunction among patients with normal sexual hormones. The mechanism between SCH and erectile dysfunction have not been clearly demonstrated. However previous study has reported that the elevated TSH concentration accompanied with sublinical hypothyroidism is related to ED.^9^ TSH elevation may reduce Luteinizing Hormone (LH) biological activity and inhibit the secretion of Gonadotropin-releasing Hormone (GnRH), and those two hormones are important to human sexual activity.^15^ In addition elevated TSH concentration is found to be associated with endothelial dysfunction which reduces the formation and availability of nitric oxide (NO).^16^ It is well-known that NO plays important roles in the relaxation of corporal smooth muscle and vascular system to attain and sustain erectile function.^17^-^19^ The systemic and also the partial dysfunction caused by TSH elevation lead to erectile dysfunction in males with SCH. Moreover this study found that ED patients with SCH had relatively lower FT4 concentrations than the controls with euthyroidism, although serum FT4 levels of all those males were within the reference range. Serum levels of FT4 are correlated with erectile dysfunction and L-thyroxine treatment restores erectile function.^7^ Previous study has reported that thyroid hormones receptors are expressed both in penis corporal cavernosa endothelial and smooth muscle cells,^20^ and supplementation of L-thyroxine could improve function of endothelial and smooth muscle cells.^7^,^21^ SCH is much common in males with ED, and treatment of which may improve erectile function. Screening for thyroid dysfunction in men presenting with ED is recommended.

Moreover this study found that PRL was significantly elevated in ED with SCH than the healthy males, even though serum PRL concentrations of all those males were within the reference range. Previous study has reported that serum PRL concentration is positively correlated with TSH level, and PRL is synchronously increasing with TSH elevation in SCH.^22^ The elevated concentration of PRL may be resulted from a compensatory increase of thyrotropin-releasing hormone (TRH) in response to thyroid function failure in SCH.^23^ It is well-known that PRL has important roles in modulating hypothalamic-pituitary-gonad axis function, and GnRH pulsatility.^8^,^24^ The remarkable PRL elevation has been demonstrated to adversely affect sexual activity and leads to impaired libido, and erectile dysfunction.^25^ Hence the relative PRL increase accompanied with TSH elevation in SCH patients may be another factor to affect erectile function, however this observation need to be studied further to confirm this observation..

In further we compared the IIEF-5 scores among ED patients, whereas no significant of IIEF-5 scores were found between males with SCH and with euthyroidism. SCH is not associated with the severity of ED among ED males. More than one study have showed that the scores of erectile dysfunction are correlated with reduced FT4 levels, and L-thyroxine supplementation could restore erectile dysfunction in patients with overt hypothyroidism.^7^,^26^ Hence the severity of impotence may be associated with degree of thyroid failure. Subclinical hypothyroidism, merely the mild thyroid failure, may adversely affect erectile function but not enough to affect the severity. Longitudinal and prospective studies are further needed to investigate this question. However given the so high prevalence of subclinical hypothyroidism not merely in ED patients but also in populations, it is still necessary to enhance screening for mild thyroid failure in men presenting with ED.

In conclusion, this study found that SCH was not uncommon in ED males, however there were no significantly differences in IIEF-5 scores between ED with SCH and with euthyroidism. The results indicated that SCH may be associated with ED but not with the severity of ED. In clinical practice, screening for thyroid dysfunction in men presenting with ED is recommended.

### Authors’ Contribution

**DC and YY** performed the project, analyzed the data and wrote the manuscript.

**QD** helped to design the project and analyzed the data.

**HH and HT** conceived, designed the project and revised the manuscript.
